# Immunoaffinity Intact Top-Down Mass Spectrometry for Quantification of Neuron-Specific Enolase Gamma, a Low-Abundance Protein Biomarker

**DOI:** 10.1021/acs.analchem.4c04677

**Published:** 2024-12-23

**Authors:** Sebastian A. H. van den Wildenberg, Sylvia A. A. M. Genet, Maarten A. C. Broeren, Joost L. J. van Dongen, Maxime C.M. van den Oetelaar, Luc Brunsveld, Volkher Scharnhorst, Daan van de Kerkhof

**Affiliations:** 1 Laboratory of Chemical Biology, Department of Biomedical Engineering, Eindhoven University of Technology, Eindhoven 5600 MB, The Netherlands; 2 Clinical Laboratory, Catharina Hospital Eindhoven, Eindhoven 5623 EJ, The Netherlands; 3 Expert Center Clinical Chemistry Eindhoven, Eindhoven 5600 MB, The Netherlands; 4 Clinical Laboratory, Máxima Medical Center Eindhoven, Veldhoven 5504 DB, The Netherlands

## Abstract

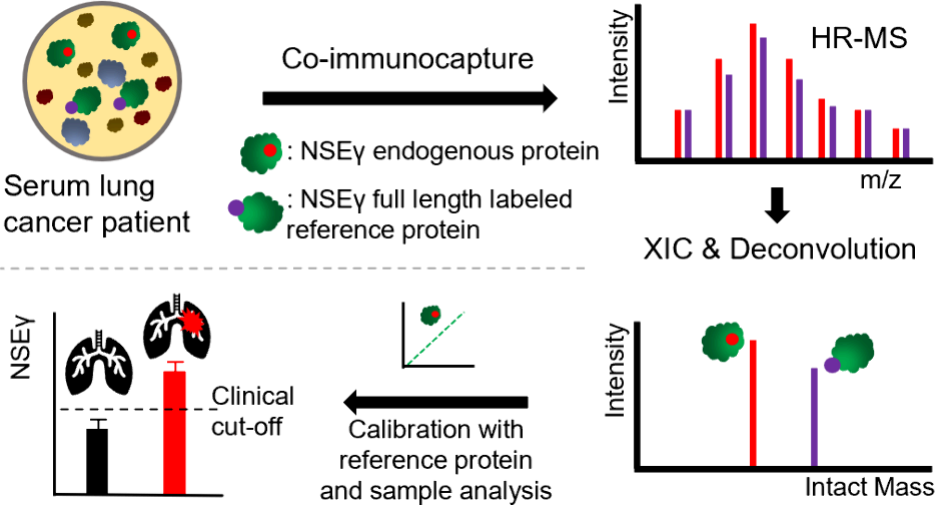

Quantification of intact proteins in serum by liquid chromatography high-resolution mass spectrometry (HRMS) may be a useful alternative to bottom-up LC-MS or conventional ligand binding assays, due to reduced assay complexity and by providing additional information, such as isoform differentiation or detection of post-translational modifications. The 47.2 kDa lung cancer tumor marker neuron-specific enolase γ (NSEγ) was quantified in a clinically relevant concentration range of 6.25 to 100 ng/mL in NSE-depleted human serum using magnetic bead immunoprecipitation coupled to LC-high-resolution quadrupole-time-of-flight MS. The novelty of the described approach is in the combined setup of immunoaffinity extraction and the use of a full-length NSEγ calibrator and labeled NSEγ internal standard (IS) to reliably quantify the post-translationally acetylated form of this protein tumor marker in a top-down proteomics workflow. Isolation parameters and quantification using deconvolution and reconstructed extracted ion chromatograms were evaluated, and the development of a suitable liquid chromatography method was demonstrated. Various validation parameters were determined using both quantification methods, both showing acceptable performance. Additionally, deconvolution-based quantification enabled an accurate mass determination. The developed method was compared to a commercially available ECLIA and showed good correlation in sera of patients suspected of lung cancer. This assay may form the starting point for the development of a reference method for the standardization of immunoassays.

## Introduction

Proteins in biofluids are mostly quantified using immunoassay-based methods such as enzyme-linked immunosorbent assays (ELISAs) or electrochemiluminescence immunoassays (ECLIAs). Because these immunoassays are known to be poorly standardized, leading to significant variation between manufacturers, reagents, and reagent lots, there is a strong need for improving metrological traceability using reference measurement procedures (RMPs).^1,2^ By convention, chromatographic methodology with mass spectrometric detection (LC-MS) has been used as the most promising RMP.^3,4^

Bottom-up LC-MS assays are the most used LC-MS assays in bioanalysis, in which one or multiple peptides, produced after proteolytic digestion of the protein, are selected as representative for the intact protein, followed by quantification using selective tandem mass spectrometry (MS)-based methods.^5^ Using this approach, peptides carrying a post-translational modification (PTM) are mostly avoided as this complicates the method development and the production of reference materials and internal standards.^6^ The downside of this signature peptide selection is that valuable information about the protein is thus lost, as the presence of isoforms and PTMs may carry valuable pathophysiological information.^7−10^ Also, quantification of isoforms requires careful selection of signature peptides^11,12^ and the development of such peptide-centric isoform specific methods requires additional development steps and is time-consuming.^11,13−15^

Different from bottom-up or middle-down based methods, (intact) top-down MS analyzes the full protein, retaining information both for different isozymes and for PTMs.^16−21^ Top-down strategies using liquid chromatography (LC) HRMS have been gaining popularity for the analysis of intact proteins as an alternative for bottom-up analysis, specifically for the characterization of protein isoforms.^15,16,22^ However, quantification using top-down approaches has its own challenges.^23^ Intact MS requires high-resolution (HR) MS, such as time-of-flight (ToF), FT-ICR, or Orbitrap mass analyzers,^24,25^ instruments that are not yet widely available in routine clinical laboratories.^26,27^ Analytical sensitivity is also relatively low because of the multiple charge states of large proteins and additional ion suppression due to matrix effects.^28^ For intact top-down MS methodology to be applied in clinical diagnostics, the required fully characterized and commutable reference materials and full-protein-labeled internal standards pose a significant challenge due to the complexity of the protein analytes. Next to that, no standardized method for this type of data integration is currently used. Data integration can be performed in various ways, such as full scan followed by quantitative deconvolution, quantification based on reconstruction of extracted ion chromatograms (XIC), or even more targeted top-down MS/MS measurements.^29^

Neuron-specific enolase (NSE) is a biomarker composed of αγ- and γγ-dimers. The γ-isozyme is predominantly expressed in neurons and neuroendocrine cells, including neuroendocrine tumors such as small-cell lung cancer (SCLC).^30,31^ NSE has been reported to be a promising tumor marker for the diagnosis and follow-up of SCLC.^32^ A bottom-up LC-MS assay has already been developed to quantify this biomarker.^11^ However, the development of an intact top-down LC-MS method could lead to a simplified method for the possible incorporation of PTMs in the analysis. This research focuses on NSEγ, a 47.2 kDa, 434 amino acid long protein, due to specificity for neurons and neuroendocrine cells (UniProt accession number P09104).^31^ With this top-down LC-MS method, we quantified the acetylated form of NSEγ, likely a PTM, which we described in previous work.^33^ Normally, serum NSE concentrations are in the low nanogram per milliliter (nM) range. Elevated concentrations of NSE are linked to lung cancer with a cutoff value of ≥25 ng/mL.^32^

To achieve absolute quantification of NSEγ, we aimed to first develop an immunoaffinity enrichment assay using magnetic beads to isolate and purify the low-abundance protein tumor biomarker; second optimize the chromatographic separation between the biomarker and interfering compounds; third produce and use an internal standard to perform a reliable immunoaffinity isolation and quantitative determination; and fourth evaluate multiple quantification approaches based on quantitative deconvolution and reconstructed XICs and their subparameters, such as the amount of selected charge states and isolation windows.

**Figure 1:**
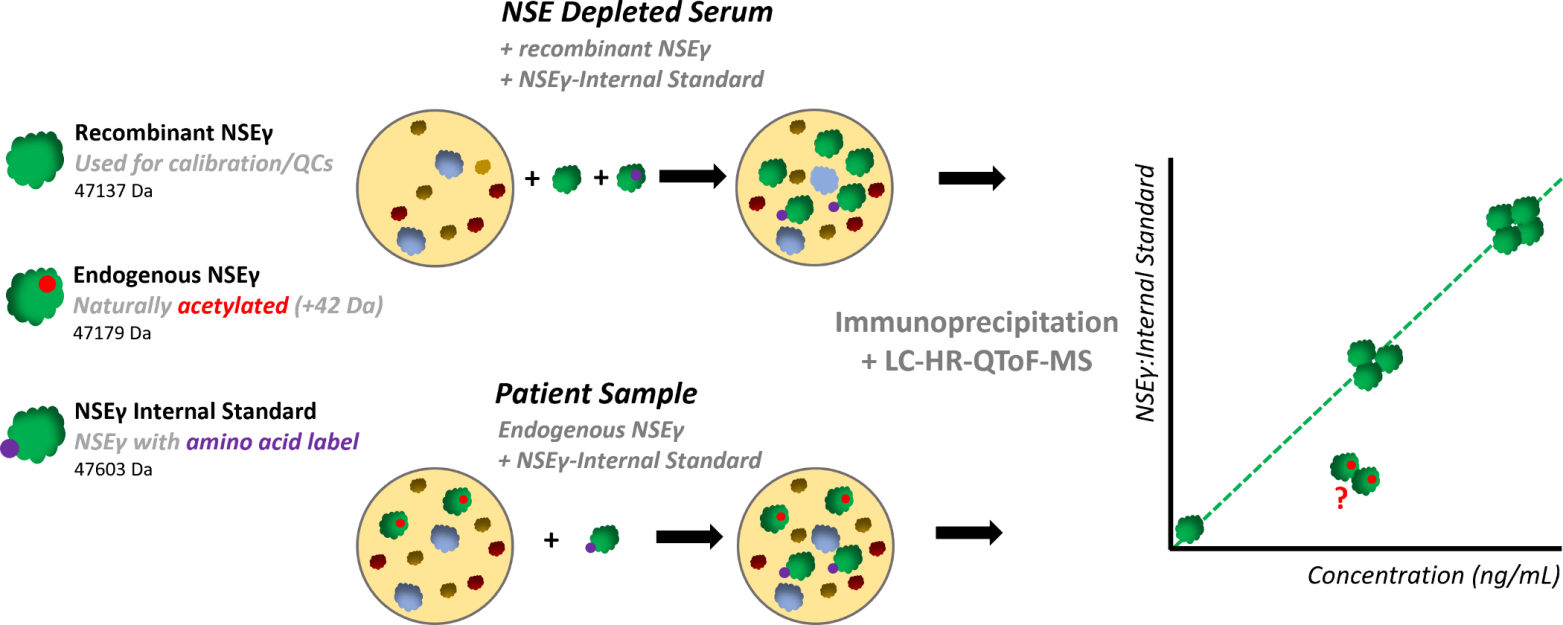
Endogenous (acetylated) NSEγ quantification is performed by spiking NSEγ depleted serum with increasing concentrations of recombinant NSEγ and the NSEγ internal standard (IS) to obtain a calibration curve based on the recombinant NSEγ:NSEγ-IS-ratio. The obtained calibration curve is used to back calculate the endogenous (acetylated) NSEγ concentration based on the endogenous (acetylated) NSEγ:NSEγ-IS-ratio.

The method uses protein G-labeled Dynabeads magnetic beads that are functionalized and cross-linked with monoclonal anti-NSEγ antibodies for protein isolation and purification, followed by UPLC coupled to HR-QToF-MS analysis. The presented method relies on the use of internal standard calibration using a reference protein with an extra (amino acid) mass label as internal standard (IS) and NSEγ-IS (47,603 Da) and recombinant NSEγ (47,137 Da) as calibrators to quantify endogenous acetylated NSEγ (47,179 Da).^33^ Both recombinant or endogenous NSEγ are co-captured using an antihuman NSE antibody via magnetic bead immunoprecipitation followed by intact protein quantification using LC-HR-QToF-MS, as displayed in Figure 1.

The novelty of the described approach is in the combined setup of immunoaffinity extraction and the use of an engineered full-length protein calibrator and a labeled internal standard to reliably quantify a complex and low-concentration protein tumor marker. This is the first validated top-down method that can potentially be used in the diagnosis of lung cancer. In the field of clinical laboratory analysis, there is also a great need of such assays to be used as a reference measurement procedure to standardize immunoassays. The presented methodology does not require tryptic digestion of proteins as applied in bottom-up methods and thus has great potential as a reference measurement procedure because it can improve precision, sensitivity, selectivity, and robustness.

## Materials and Methods

### Chemicals and Reagents

An anti-human NSE 9601 SPTN-5 monoclonal mouse antibody was obtained from Medix Biochemica (lot: 0046841) (Espoo, Finland). Protein G Dynabeads and BS(PEG)_5_ were purchased from Thermo Fisher Scientific (Waltham, Massachusetts, USA). LC-MS-grade mobile phase solvents were obtained from Biosolve (Valkenswaard, The Netherlands).

### Reference and Internal Standard Protein Expression

Recombinant NSEγ and NSEγ-IS were expressed in *Escherichia coli* BL21 (DE3) cells with a pET28a(+) plasmid (GenScript Biotech UK Limited, Oxford, United Kingdom). Both proteins were expressed and purified following standard procedures using Ni-NTA column purification, corresponding tag cleavage using SUMO-hydrolase or thrombin protease, and reversed Ni-NTA column purification to remove cleavage enzymes and cleaved purification tags (S1). Sequences before and after tag cleavage are displayed in S2. Exact mass and purity were confirmed using high-resolution high-resolution QToF LC-MS (S4). Additionally, LC-UV measurements were performed for both proteins (S3). Both purified proteins were dissolved and aliquoted in 20 mM Tris–HCl at pH 7.4, 150 mM NaCl, and 0.5 mM TCEP and stored at −80 °C until use. Recombinant NSEγ and NSEγ-IS had respective formulas of C_2089_H_3308_N_568_O_650_S_13_ and C_2106_H_3347_N_575_O_654_S_13_, with corresponding molecular weights of 47,137.07 and 47,602.66 Da, respectively. The theoretical NSEγ-masses were calculated accurately using the atomic weights of common elements in proteins (land plants with the C3 metabolic process and other organic sources): C: 12.01079, H: 1.007968, N:14.00669, O:15.99937, and S: 32.0639 Da.^34^

### Antibody Coupling to Protein G-Functionalized Magnetic Beads

The coupling procedure was performed as published in previous work^11,33,35^ using LoBind Falcon tubes, according to the following general procedure for one equivalent (*n*): 8.33 μL of protein G-labeled Dynabeads (Thermo Fisher Scientific, Waltham, Massachusetts, USA) were washed twice in 250 μL of conjugation buffer (100 mM sodium phosphate, 70 mM NaCl, 0.05% Tween 20 (v/v), pH 8.0). The supernatant was removed, and 250 μL of 8 μg/mL Anti-h NSE 9601 dissolved in conjugation buffer was added to the beads. After 30 min of incubation with rotation, 1.25 μL of 5 mM BS(PEG)_5_ dissolved in cold conjugation buffer was added (final concentration 25 μM) and incubated for 30 min with rotation. The reaction was quenched by adding 25 μL of 1 M Tris–HCl, pH 7.5, for 15 min with rotation. The supernatant was removed, and unbound antibody was eluted by adding 50 μL of elution buffer (1 M NaSCN in 100 mM Tris–HCl, pH 7.5) for 5 min with rotation. The beads were then washed twice with 250 μL of PBS, 0.1% Tween 20, pH 7.4, and subsequently stored in this buffer at 4 °C until further use. For large-scale experiments, volumes were multiplied by the number of equivalents (*n*).

### Immunoprecipitation

For all serum samples described in this study, 50 μL of the prepared antibody coupled beads were transferred to 1.0 mL of sample in a 2 mL LoBind Eppendorf tube. Samples were incubated for 2 h at room temperature with 20 rpm rotation frequency, and after removal of the supernatant, the beads were successively washed two times with 200 μL of PBS, pH 7.4; after the first wash, the bead suspension was transferred to a new 2 mL LoBind Eppendorf tube. The wash solvent was removed, and the beads were then resuspended in 50 μL of elution buffer (Milli-Q/ACN (80:20) + 1.0% FA) and incubated in a shake incubator at 1200 rpm for 5 min at room temperature. Successively, the magnetic beads were removed, and the supernatant was transferred to LC-MS vials for analysis.

### Preparation of NSE-Depleted Serum

NSE-depleted serum was prepared as described previously.^11^ Whole blood of 10 healthy volunteers was collected in 8.5 mL Vacutainer SST II Advance Plus Blood Collection Tubes (Becton Dickinson, Franklin Lakes, New Jersey, USA) during routine venipuncture. The sera were obtained within 1 h after collection by centrifugation for 10 min at 2683*g* at 20 °C and subsequently pooled. Depletion of NSE from serum was performed in six incubation steps using the prepared antibody coupled beads. After incubation, the serum supernatant was collected, and the captured NSE was eluted using 1 M NaSCN. The beads were re-equilibrated prior to renewed addition to the serum supernatant. The remaining NSE concentration was checked four times, after each consecutive cycle, using the bottom-up LC-MS/MS procedure described previously^11^ and an ECLIA assay using an Elecsys analyzer (Roche Diagnostics, Rotkreutz, Switzerland). The NSE concentration was found to be under the detection limit after four depletion cycles.

### LC-HR-QToF-MS

Analysis of the eluted proteins was performed on a Waters Xevo G2-XS HR QToF coupled to an ACQUITY UPLC I-class binary solvent manager and ACQUITY UPLC Sample Manager-FL (Milford, Massachusetts, USA). An Agilent Polaris C18-A 2.0 × 100 mm, 3 μm, Agilent Polaris C18-A 2.0 × 150 mm, 3 μm (Agilent Technologies, Middelburg, The Netherlands) and Thermo Fisher MAbPAC 2.1 × 100 mm column (Thermo Fisher Scientific, Waltham, Massachusetts, USA) were used for chromatographic separation with a flow of 0.3 mL/min and a column temperature of 60 °C for the Polaris columns and 80 °C for the MAbPAC column. The mobile phases consisted of (A) 0.1% FA in Milli-Q water (MQ) (<5 ppb total organic carbon (TOC) and (B) 0.1% FA in ACN. The gradient in minutes per percentage of mobile phase B was set as follows (all displayed as % v/v): 0.0–11.5 min (25–50% B), 11.5–12.5 min (50–75% B), 12.5–13.0 min (75% B), 13.0–13.1 min (75–25% B), 13.1–15.0 min (25% B). Electrospray ionization (ESI) was operated in positive ionization mode. MS settings were set as follows: capillary voltage 0.8 kV, sampling cone 40, source offset 80, source temperature 120 °C, desolvation temperature 450 °C, cone gas 10 L/h, and desolvation gas 1000 L/h. Prior to the batch analysis, the instrument was calibrated using phosphoric acid over a range of 100 to 2000 M/z. LeuEnk (556.27 M/z) was used as LockSpray (calibration) during each measurement at 0.5 min intervals. All LC-MS parameters were controlled using MassLynx software (Waters, version 4.2).

### Data Analysis

Quantitative deconvolution was performed by selecting the five most abundant peaks in the full-scan spectrum. MaxEnt1 deconvolution was performed using MassLynx software (Waters, version 4.2) over a range of 46,000 to 49,000 Da. A simulated isotope pattern was used with a spectrometer blur width of 0.330 Da as a damage model. Left and right minimum intensity ratios were set at 33%. Completion was set to iterate to convergence. For optimization purposes, resolution was varied between 0.1 and 2.0 Da/channel. After deconvolution, peaks were centered using the described settings, and peak area was obtained for quantification. XICs were produced by isolating 1 up to 10 charge states from the protein, ranging from the 49+ to 58+ protein charge states with isolation windows starting from 0.1 to 5.0 Da. For quantification purposes, peak integration was performed using the obtained reconstructed XICs.

### Calibration and Validation

The intra- and interassay precision and accuracy of NSEγ quantification were investigated for 3 days (*n* = 5, for each day). Recombinant NSEγ was spiked at 5.0 (lowest QC), 15.0 (low QC), 40 (medium QC), and 80 (high QC) ng/mL in NSE-depleted serum (NDS) samples. The intra- and interassay precision and accuracy were analyzed at the four QC levels according to the calculations by Krouwer and Rabinowitz.^36^ Data were expressed as coefficients of variation (% CV) and accuracy (%). Calibration standards were prepared in NDS at 6.25, 12.5, 25.0, 37.5, 50.0, 75.0, and 100 ng/mL recombinant NSEγ, combined with IS at 10 ng/mL. Additionally, blank (no NSEγ, IS only) and double-blank samples (no NSEγ, no IS) were analyzed. For each analytical run, XIC peak areas of NSEγ with IS corrections were plotted versus the nominal protein concentrations. Additionally, deconvoluted peak areas of NSEγ with IS corrections were plotted versus the nominal protein concentrations. The nominal concentrations of the calibration standards were fitted using 1/*x* weighted regression using GraphPad Prism (version: 10.2.1 (395), GraphPad Software LLC, San Diego, California, USA). The limit of detection (LoD) and limit of quantification (LoQ) were calculated according to the following formulas: LoD: 3.3 × standard error of *Y*-intercept (Sy.x)/best-fit slope (B1), LoQ: 10 × standard error of *Y*-intercept (Sy.x)/best-fit slope (B1). The carryover was determined by integrating the peak area in a blank sample after the highest calibration sample of 100 ng/mL. Limits for carryover are ≤20% of the LLoQ signal and ≤5% of the IS signal and are based on the EMA guidelines.^37^ The analyte recovery was determined by spiking a serum sample before and after immunoprecipitation. Matrix effects were determined by spiking anonymized residual serum of six individuals with 50 ng/mL NSEγ, and the differences between the measured values and nominal concentrations were calculated and expressed as percentage deviation (%).

### Comparative Study

The endogenous (acetylated) NSEγ concentrations in serum of 23 selected study participants from lung marker study NL9146, ICTRP Search Portal (who.int)) described elsewhere,^11,32,38−41^ ethically approved by the Medical Research Ethics Committees United (NL58985.100.16), were quantified using a recombinant NSEγ calibration curve. Eight no malignancy, seven nonsmall cell lung cancer (NSCLC), and eight small-cell lung cancer (SCLC) samples were measured using both a commercially available ECLIA (Cobas platform e801, Roche Diagnostics, Rotkreuz, Switzerland) and the developed (XIC) MS method and compared using a Bland-Altman analysis to determine the average difference between the ECLIA and MS method. Statistical analyses were performed in GraphPad Prism (version 10.2.1, GraphPad Software LLC, San Diego, California, USA).

## Results

### Top-Down Quantitative Data Integration

**Figure 2:**
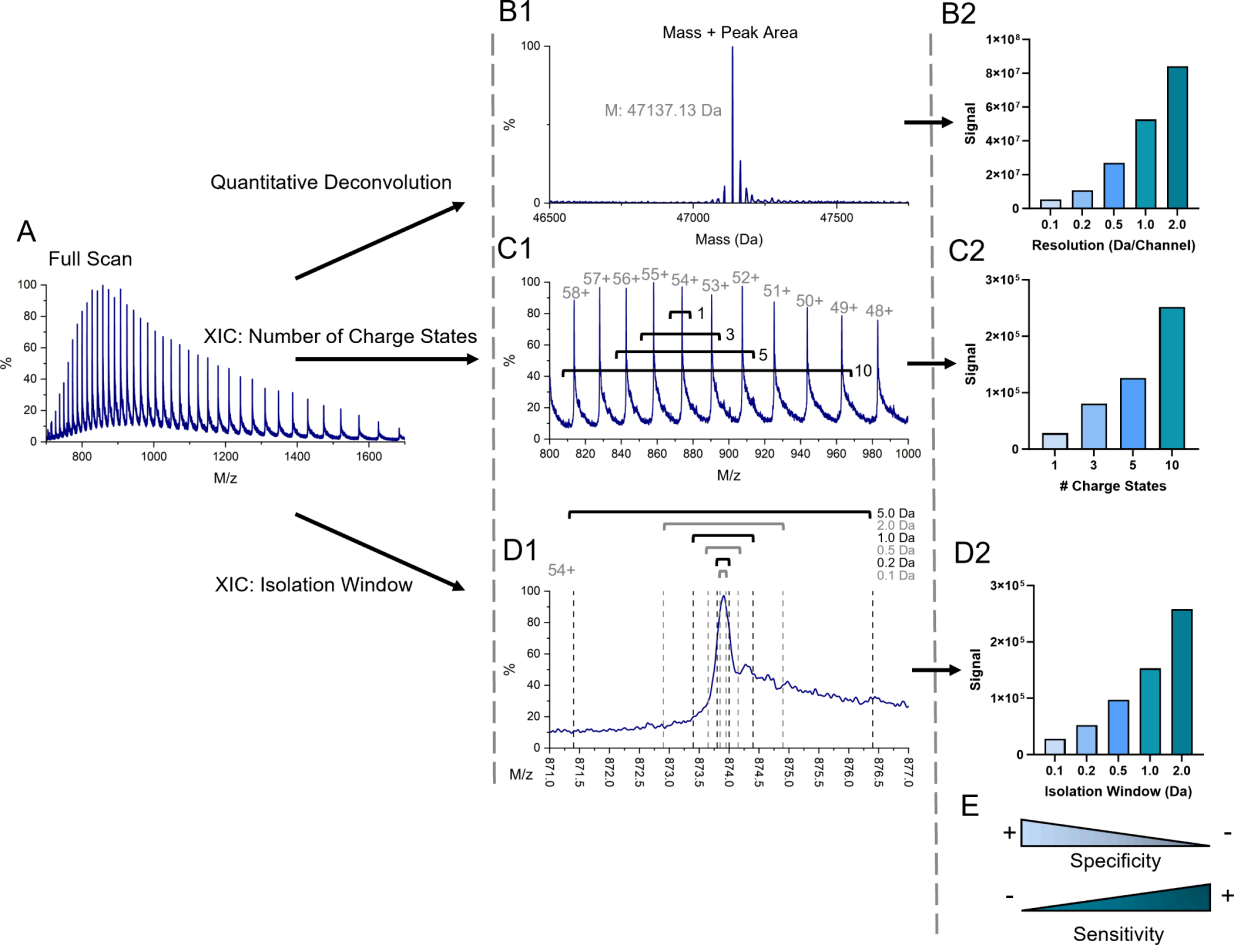
Full-scan top-down quantitative data integration of recombinant NSEγ; full-scan data (A) and quantitative deconvolution (B1, B2) with 0.1–2.0 Da/channel resolution for peak intensity determination. Reconstructed extracted ion chromatograms (XICs) using 1 to 10 charge states for peak area determination (C1, C2). Isolation windows from 0.1 to 2.0 Da for XIC determine the integrated peak width (D1, D2). Different settings influence the specificity and sensitivity of the data integration (E).

Two different intact protein top-down MS quantification approaches were investigated, based on peak integration from quantitative spectrum deconvolution and reconstructed XICs. Figure 1A shows the charge-state envelope of recombinant NSEγ, resulting from a full-scan analysis over a range of 500 to 2000 *m*/*z*. This full-scan data was processed via the two selected methods. A zero-charged deconvoluted mass spectrum is displayed in Figure 2B1, with the effect of deconvolution resolution on the peak area displayed in Figure 2B2. Selected resolutions were 0.1, 0.2, 0.5, 1.0, and 2.0 Da/channel. Reduced resolution (corresponding to increased Da/channel) resulted in higher analytical sensitivity, but with theoretically reduced specificity as a result. This potentially leads to increased risk of interferences when analyzing biological samples. Using deconvolution, it is possible to obtain the intact mass of the protein (recombinant NSEγ 47,137.13 Da), next to the peak intensity determination for quantification.

Figure 2C1 shows a series of the most abundant charge states of the protein (charge states 48+ to 58+) in a *m*/*z* range of 800 to 1000. Respectively, 1, 3, 5, and 10 charge states were selected to reconstruct the XIC chromatograms. After integration of the obtained reconstructed XIC chromatograms, the peak area was found to increase when a higher number of charge states were included (Figure 2C2). However, the specificity will likely decrease when more charge states are used.

Figure 2D1 shows the proteins’ 54+ charge state with XIC isolation windows starting from 0.1 up to 5.0 Da. A small isolation window results in a highly specific isolation of the target *m*/*z*. In the case of NSE, the smallest window indeed resulted only in a partial coverage of the charge-state peak, resulting in a lowered intensity of the reconstructed peak. A larger isolation window resulted in a higher intensity and thus more sensitivity. Figure 2D2 shows the results for all of the isolation windows.

Both quantitative deconvolution with 0.1 Da/channel and XIC peak reconstruction using five charge states with a 0.1 Da isolation window were used for further calibration and validation experiments in this study. The two methods were both used to evaluate their applicability in clinical assays, since no standard quantification method methodology is currently available in the field of intact protein quantification using HRMS.

### Liquid Chromatographic Method Development

**Figure 3:**
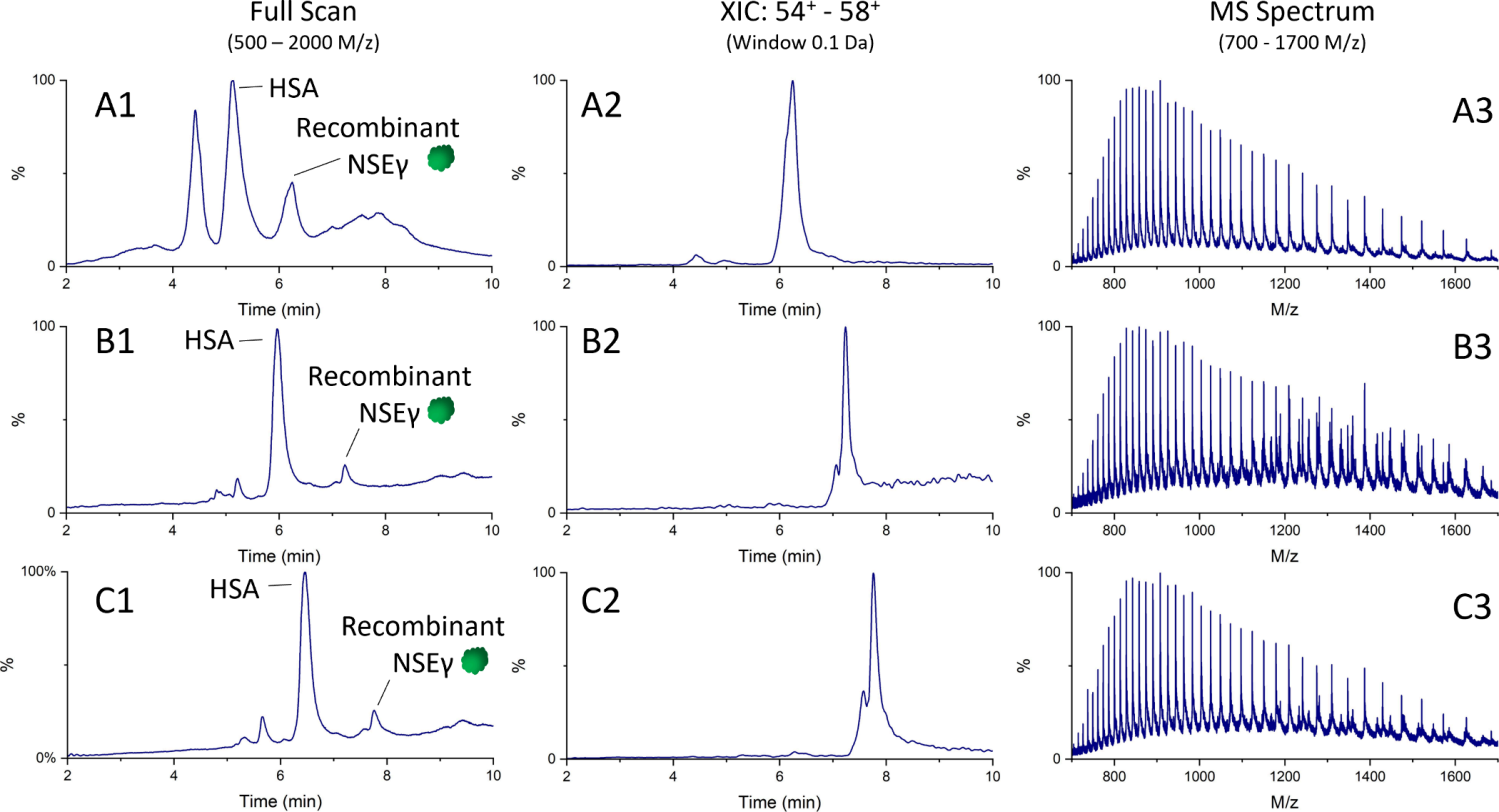
Full-scan chromatograms (panel 1), reconstructed extracted ion chromatograms (XIC) (panel 2), and MS spectra (panel 3) using the respective MAbPAC (A), Polaris 3 C18 100 mm (B), and Polaris 3 C18 150 mm (C) analytical columns for LC-method development of recombinant NSEγ isolated using immunoprecipitation from NSE-depleted human serum with nonspecifically co-captured human serum albumin (HSA) as the main interfering peak.

Three analytical columns were studied for the separation of immunoprecipitated recombinant NSEγ, from any other proteins that were coprecipitated and eluted from the magnetic beads. Full-scan chromatograms of the analyses using a MabPAC column and Polaris 100 and 150 mm columns are displayed in Figure 3A1,B1,C1. Chromatographic resolution was calculated using the NSEγ target peak and interfering human serum albumin (HSA) peak labeled with “HSA” (Figure 3A1,B1,C1). The chromatographic resolutions were 2.6, 4.2, and 4.4 for the MabPAC, Polaris 100 mm, and Polaris 150 mm columns, respectively (Table 1). The signal-to-noise ratios were determined using the MassLynx software from the reconstructed XICs (Figure 3A2,B2,C2) and were found to be 713, 247, and 380 for the MabPAC, Polaris 100 mm, and Polaris 150 mm columns, respectively (Table 1). Although the highest resolution was found using the Polaris 150 mm column, representing better chromatographic separation, the MabPAC column was selected for further use due to the higher signal-to-noise ratio, enabling higher analytical sensitivity.

**Table 1:** Analytical Performance Based on Chromatographic Resolution and Signal-to-Noise Ratio of the MAbPAC, Polaris 3 C18 100 mm, and Polaris 3 C18 150 mm Columns for the Analysis of Recombinant NSEγ Isolated from NSE-Depleted Human Serum Using Immunoprecipitation

**panel**	**column**	**retention time recombinant NSEγ (min)**	**chromatographic resolution (HSA/NSEγ)**	**signal-to-noise ratio (RMS)**
A	MAbPAC	6.27	2.6	713
B	Polaris 100 mm	7.23	4.2	247
C	Polaris 150 mm	7.76	4.4	380

### Calibration and Validation

**Figure 4:**
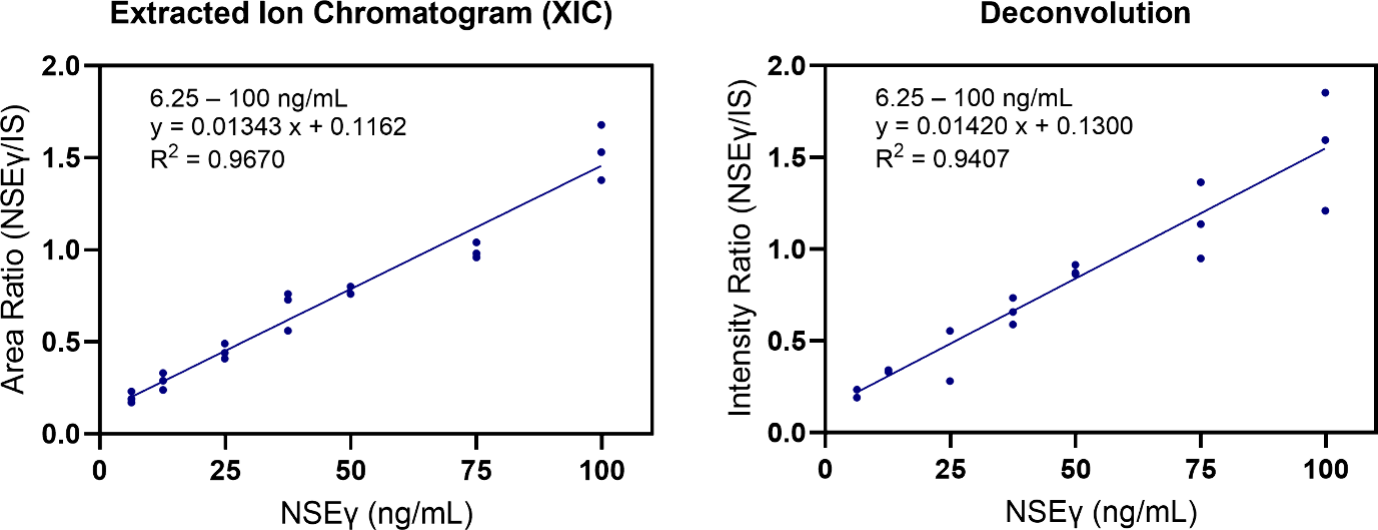
Calibration of recombinant NSEγ in NSEγ-depleted serum (NDS) over a range of 6.25 to 100 ng/mL (3 days). Peak integration using reconstructed extracted ion chromatograms (XIC) (left) and quantitative deconvolution (right) with weighted linear regression (1/*x*). The recombinant NSEγ:NSEγ-internal standard ratio is plotted versus the NSEγ concentration (ng/mL).

A calibration curve using NDS spiked with recombinant NSEγ was analyzed on three subsequent days using weighted linear regression (1/*x*). Linearity was achieved within the range of 6.25 to 100 ng/mL NSEγ, with *R*^2^ values of 0.9670 and 0.9407 using reconstructed XICs and quantitative deconvolution, respectively (Figure 4). Both LoD and LoQ were calculated using the standard error of the *Y*-intercept and the best-fit slope. The calculated LoD and LoQ using reconstructed XICs were 3.5 and 10.6 ng/mL, respectively. The calculated LoD and LoQ using quantitative deconvolution were 4.2 and 12.8 ng/mL, respectively. Additionally, the carryovers were found to be 9.2% of the LLoQ (acceptable limit: ≤20%) and 1.7% of the IS (acceptable limit: ≤5%) and were within the acceptance limits. NSEγ recovery was found to be 80.6%. The within-run and between-run precision and overall accuracy were determined during 3 days, for which at each day four quality control levels were analyzed fivefold (Table 2). Using quantitative deconvolution, no precision could be determined at the lowest-quality control levels, due to the lack of analytical sensitivity using this methodology. Some between-run precision values were found to be 0%, due to the calculated between-run mean squares being smaller than the within-run mean squares (Table 2). The average deconvoluted recombinant NSEγ mass during calibration and validation was 47,137.30 Da with an average MaxEnt error of ±0.61 and 47,602.79 Da with an average MaxEnt error of ±0.36 Da for the NSEγ-IS (with 47,137.07 and 47,602.66 Da as respective calculated protein mass). To examine potential matrix effects, residual serum of six healthy individuals was spiked using 50 ng/mL recombinant NSEγ, and the concentrations were determined using a recombinant NSEγ calibration curve after subtracting the endogenous NSEγ signal from the corresponding unspiked sample. All measured values were found to be within acceptance limits (≤+–/15%). The average matrix effect was found to be −3.1% (S4).

**Table 2:** Accuracy and Precision Results for Immunoaffinity Precipitation Followed by LC-HR-QToF-MS Quantification of Recombinant NSEγ Based on Quantitative Deconvolution and Reconstructed Extraction Ion Chromatograms (XICs) (3 Days, *n* = 5)

			**accuracy** (%, bias)	**precision** (%, CV)	
**quantification method**	**QC**	**concentration** (ng/mL)	**overall**	**within run**	**between run**
**deconvolution**	lowest	5	N.D.	N.D.	N.D.
	low	15	107%	26%	0.0%^a^
	medium	40	108%	7.6%	13%
	high	80	101%	12%	8.0%
**XIC**	lowest	5	131%	27%	0.0%^a^
	low	15	114%	9.2%	0.0%^a^
	medium	40	109%	8.5%	8.5%
	high	80	106%	6.8%	3.8%

a Calculated between run mean squares being smaller than the within-run mean squares.

### Comparative Study

**Figure 5:**
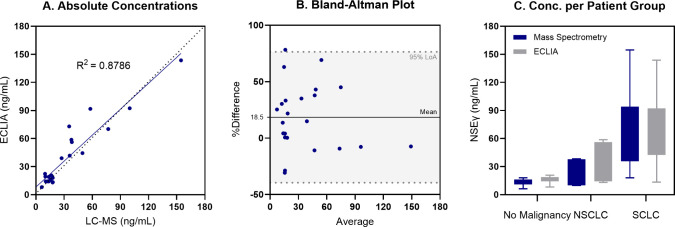
Obtained concentrations using the ECLIA method vs the developed MS method with reconstructed XIC data integration with *R*^2^ = 0.8786 (*n* = 23) (A). Bland-Altman analysis of the ECLIA and MS method compared with the mean difference and 95% limits of agreement (LoA) displayed (*n* = 23)) (B). Boxplots of the endogenous NSEγ concentration in patients without malignancy (*n* = 8), NSCLC patients (*n* = 7), and SCLC patients (*n* = 8) (C). Endogenous NSEγ concentrations were determined by using a recombinant NSEγ calibration curve.

Serum samples of eight patients without malignancies, seven with NSCLC, and eight with SCLC were analyzed with the developed immunoaffinity intact mass analysis MS method with XIC data integration. All the data (*n* = 23) were compared to a commercially available ECLIA. The obtained NSEγ concentrations using the ECLIA method (*y*-axis) were plotted against the obtained concentration using the MS method followed by reconstructed XIC data integration (*x*-axis) (Figure 5A). The measured concentrations showed agreement between the two methods with an *R*^2^ of 0.8786. The analytical bias between the methods was determined to be 18.5% (95% LoA: −39.5 to 76.5), as displayed in the Bland-Altman plot (Figure 5B). The range of measured NSE concentrations in sera of patients without malignancy, NSCLC patients, and SCLC patients were comparable for both assays (Figure 5C).

**Figure 6:**
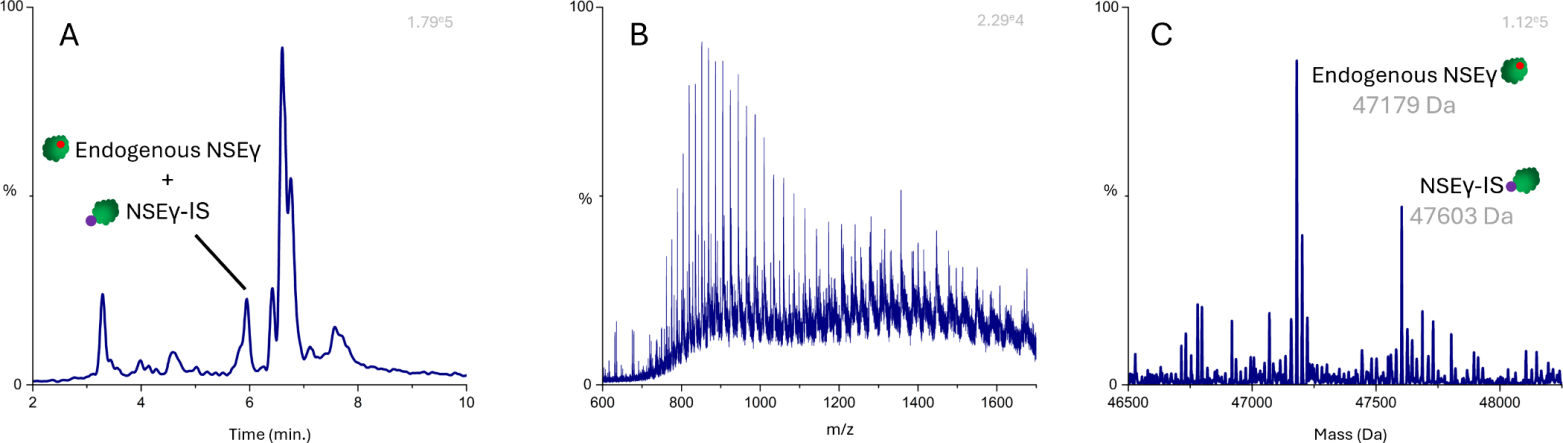
Typical data from patient sample analysis. XIC after immunoaffinity purification followed by intact mass analysis (A). Mixed MS spectra of endogenous NSEγ and NSEγ-IS (B). MaxEnt 1 deconvoluted MS spectra of endogenous NSEγ (47,179 Da) and NSEγ-IS (47,603 Da) (C).

Figure 6 shows typical data for an analyzed patient sample after immunoaffinity purification followed by LC-HR-QToF-MS. Endogenous NSEγ was found to have the same elution times compared to recombinant NSEγ and NSEγ-IS. Although specific monoclonal anti-NSEγ antibodies were used for immunoprecipitation, nonrelated compounds are also isolated and detected (Figure 6A). Figure 6B shows a spectrum in the range of 600 to 1700 *m*/*z* with both endogenous NSEγ, NSEγ-IS, and other interfering compounds. Lastly, Figure 6C shows the deconvoluted masses of both isolated endogenous NSEγ and NSEγ-IS.

## Discussion

This work describes a robust intact top-down high-resolution MS quantification method for lung cancer tumor biomarker NSEγ, where (unacetylated) NSEγ is used to quantify endogenous (acetylated) NSEγ. The method comprises an immunoprecipitation step followed by intact mass protein quantification with a protein IS using LC with high-resolution quadrupole time-of-flight analysis. Although the analytical performance of the developed assay showed similar results compared to the ECLIA assay, future work should focus on the use of an acetylated recombinant NSEγ and an acetylated NSEγ-IS to be more representative of the endogenous acetylated NSEγ. Additionally, the use of a different IS with, e.g., stable heavy isotope ^15^N and ^13^C labels, instead of an amino acid label should be investigated.

The optimal settings of quantitative deconvolution and reconstructed XICs quantification were evaluated by varying resolution (Da/channel), the amount of used charge states, and the XIC isolation window. For quantitative deconvolution, 0.1 Da/channel was selected as the most optimal setting based on selectivity. For XIC isolation, the use of five charge states with a window of 0.1 Da isolation was selected based on sensitivity and selectivity. The selection of the amount of charge states and windows remains highly specific for each protein or biomarker. The main takeaway message is that (the amount of) charge states combined with isolation window need to be carefully selected to prevent the introduction of nonrelated peaks that might be present; other literature does not provide a clear consensus on what the best option is and options are varied within specific studies.^16,22,42,43^ When the mass spectrometric resolution is too low to separate the target from interfering peaks, deconvolution-based quantification can be a valuable alternative due to its high specificity. However, it has the downside of being a less sensitive method.

Three analytical columns were tested for their suitability to separate NSEγ from nonspecifically isolated interfering proteins after immunoprecipitation. The MAbPAC column was selected based on resolution from other chromatographic peaks and highest signal-to-noise ratio. Notwithstanding the immunoprecipitation, nonspecific protein binding of mainly HSA remains present, as indicated in Figure 3 peak “HSA”. The IA-LC method developed here allowed isolation and separation of NSEγ present in low ng/mL concentrations from HSA present at around 40 mg/mL. Future work could additionally focus on the use of lower flow-rate chromatography such as micro- or nanoflow LC, for improved separation and sensitivity and can potentially include quantification of other biomarkers, including NSEα, to determine total NSE concentration in a multiplex fashion.

Two quantification approaches were applied: quantitative deconvolution and summation of reconstructed extracted ion chromatograms. Deconvolution offers both quantitative data and can be used to confirm protein presence by accurately determining the mass, which was nicely demonstrated by using the validation runs. However, the sensitivity and precision were lower using quantitative deconvolution compared to quantification using reconstructed XICs, which could be explained by the deconvolution algorithm that was used. Peaks with relatively low intensity might not be accurately reconstructed and are possibly discarded. Also, due to the complexity of the sample, the deconvolution algorithm might not be able to distinguish the specific signal from noise. Contrarily, reconstructed XIC quantitation integrates the full signal within the specified *m*/*z* range (Figure 2D). Acceptable analytical sensitivity, accuracy, and precision were obtained over the linear dynamic range, allowing for robust NSEγ quantification. Based on the limited literature available in the field of quantitative intact protein analysis, there is currently no standard method for processing this data.^16,22,43^ Since full-scan analysis allows for both postrun data integration options, quantification using reconstructed XICs is recommended, followed by mass deconvolution for protein confirmation/identification purposes. The sensitivity (LoD) 3.5/4.2 ng/mL was higher than a commonly used ligand binding assay for NSE (0.15 ng/mL); however, the sensitivity is comparable to other intact protein quantification methods for different proteins and is more than adequate to achieve the 25 ng/mL cutoff level for lung cancer.^16,32,42^

Using the MS assay, similar NSEγ concentrations were found compared to the ECLIA. Although the antibody used in this study (Anti-h NSE 9601) has a higher affinity for both human NSE and recombinant NSE compared to the ECLIA (Anti-h NSE 18E5 and Anti-h NSE 84B10; sandwich), the developed intact top-down LC-MS assay was found to be less sensitive compared to the ECLIA.^44^ The diagnostic performances of the MS and the ECLIA in discriminating NSCLC and SCLC from benign cases were similar, although no significant conclusion could be drawn due to the limited sample size.

This newly developed intact mass analysis assay could be the starting point for the development of an RMP for the standardization of immunoassays. Standardization might help the broader clinical application of tumor marker analysis. Current immunoassays are not standardized, leading to different analytical results between the different immunoassay platforms, all with the risk of misclassification of the disease state. More reliable and cross-platform comparable data will lead to an increased clinical value of tumor marker data; this highlights the clinical need to standardize these assays using RMPs. This is demonstrated using the IA- LC-HR-QToF-MS assay developed in this work, combined with the use of well-defined reference materials such as the unlabeled reference protein and labeled internal standard protein.

In conclusion, this novel immunoprecipitation LC-HR-QToF-MS method makes use of full-length internal standards that allow for correction of variations in the complete immunoprecipitation method. Using this method, it is possible to reliably quantify endogenous NSEγ, a low-concentration cancer protein biomarker in human serum.

## Supporting Information Available

(S1) Protein expression protocol, (S2) protein sequences, (S3) expressed protein characterization LC-UV system, (S4) expressed protein characterization LC-MS system, and (S5) matrix effect (PDF)
