# Supplementary material for: Immunoaffinity Intact Top-Down Mass Spectrometry for Quantification of Neuron-Specific Enolase Gamma, a Low-Abundance Protein Biomarker
Source: Anal Chem. 2024 Dec 23;97(1):516–25. doi: 10.1021/acs.analchem.4c04677 (PMC11740178; doi:10.1021/acs.analchem.4c04677)
Supplement: Supplementary file 1 — ac4c04677_si_001.pdf [file ac4c04677_si_001.pdf]

Immunoaffinity Intact Top-Down Mass Spectrometry for Quantification of Neuron Specific Enolase Gamma, a low abundant protein biomarker – Supplementary Information

Sebastian A. H. van den Wildenberg[1, 2, 3], Sylvia A. A. M. Genet[1, 2, 3], Maarten A. C. Broeren[1, 3, 4], Joost L. J. van Dongen[1, 3], Maxime C.M. van den Oetelaar[1], Luc Brunsveld[1, 3], Volkher Scharnhorst[1, 2, 3], Daan van de Kerkhof[1, 2, 3,*].

[1] Laboratory of Chemical Biology, Department of Biomedical Engineering, Eindhoven University of Technology, 5600 MB Eindhoven, the Netherlands

[2] Clinical Laboratory, Catharina Hospital Eindhoven, 5623 EJ Eindhoven, the Netherlands

[3] Expert Center Clinical Chemistry Eindhoven, 5600 MB Eindhoven, the Netherlands

[4] Clinical Laboratory, Máxima Medical Center, Eindhoven/Veldhoven, 5504 DB Veldhoven, the Netherlands.

**Corresponding author: daan.vd.kerkhof@catharinaziekenhuis.nl and d.h.v.d.kerkhof@tue.nl*

Supporting information tabel of contents

Page S1. Supporting Information 1 – Protein Expression Protocol

Page S2. Supporting Information 2 – Protein Sequences

Page S3. Supporting Information 3 Expressed Protein Characterization LC-UV system

Page S4. Supporting Information 4 Expressed Protein Characterization LC-MS system

Page S5. Supporting Information 5 Matrix Effect

Page S1. Supporting Information 1 – Protein Expression Protocol

Standard recombinant- and internal standard- NSEγ Protein Expression Protocol

BL21 competent *Escherichia coli* cells (DE3) were transformed with the pET28a(+) vector containing the NSEγ sequence (His-SUMO-NSEγ or NSEγ-Thrombin-His). The transformed BL21 *E. coli* cells were precultured in 8 mL LB medium containing 50 µg/mL kanamycin and were grown overnight at 37 °C, 250 rpm. The preculture was transferred to 1 L TB medium (50 µg/mL kanamycin) and incubated at 37 °C, 140 rpm until an OD600 of 0.6 - 1.2 was reached. Subsequently, protein expression was induced by the addition of isopropyl β-D-1-thiogalactopyranoside (IPTG) to a final concentration of 0.1 mM, followed by overnight incubation at 18 °C, 140 rpm. Cells were harvested by centrifugation (10.000 xg, 10 minutes, 4 °C) and were resuspended in lysis buffer (50 mM HEPES pH = 8.0, 300 mM NaCl, 20 mM imidazole, 2 mM β-mercaptoethanol (BME), 1 tablet of protease inhibitors and 5-10 µL benzonase, 10 mL lysis buffer per gram of pellet). Lysate was obtained by sonication (protocol of 5 minutes, 5 seconds pulse on, 5 seconds pulse off, amplitude of 70%) using the Qsonica Q500 and the protein-containing supernatant was obtained by centrifugation at 20.000 rpm, 30 minutes, 4 °C. The supernatant was loaded onto Ni-NTA affinity column (HisTrap high performance cartridges, Cytiva), pre-equilibrated with wash buffer (50 mM HEPES pH = 8.0, 300 mM NaCl, 40 mM imidazole, 2 mM BME). After washing the column with a wash buffer, protein bound to the column was eluted with 200 mM imidazole. The eluted protein was added to a dialysis bag (10.000 Da pores), placed in 1 L dialysis buffer (25 mM HEPES pH = 8.0, 200 mM NaCl, 2 mM BME), this was done twice. For the His-SUMO-NSEγ, SUMO hydrolase was added in a 1:100 ratio (SUMO hydrolase: His-SUMO-NSEγ protein). After overnight dialysis, the mixture was loaded onto a Ni-NTA affinity column (HisTrap high performance cartridges, Cytiva), pre-equilibrated with wash buffer (50 mM HEPES pH = 8.0, 300 mM NaCl, 40 mM imidazole, 2 mM BME) and NSEγ was purified from the cleaved His-SUMO tag and the His-tag containing SUMO hydrolase. For the NSEγ-Thrombin-His, thrombin was added in a 1:100 ratio (Thrombin: NSEγ-Thrombin-His). After overnight dialysis, the mixture was loaded onto a Ni-NTA affinity column (HisTrap high performance cartridges, Cytiva), pre-equilibrated with wash buffer (50 mM HEPES pH = 8.0, 300 mM NaCl, 40 mM imidazole, 2 mM BME) and NSEγ was purified from the cleaved thrombin-His tag and the His-tag containing the thrombin cleavage site. The purified protein (tag-less-NSEγ or NSEγ from thrombin cleavage; internal standard) was again added to a dialysis bag (10.000 Da pores) and placed in 1 L dialysis buffer (25 mM HEPES pH = 8.0, 200 mM NaCl, 2 mM BME). After overnight dialysis, the dialysis bag was transferred into the final buffer (25 mM HEPES pH = 8.0, 100 mM NaCl, 0.5 mM TCEP) and dialyzed again overnight. Proteins were aliquoted and stored at -80 °C until use.

Page S2. Supporting Information 2 – Protein Sequences

Sequence Alignment of recombinant and internal standard NSEγ before and after purification tag cleavage. Alignment using Search and sequence analysis tools services from EMBL-EBI. [1]

```
NSEγ-Internal-Standard_After-Purification-Tag-Cleavage      ------------------------------------------------------------      0
NSEγ-Internal-Standard_Before-Purification-Tag-Cleavage     ------------------------------------------------------------      0
Recombinant_NSEγ-Before-Purification-Tag-Cleavage           GHHHHHHHHHHGGSDSEVNQEAKPEVKPEVKPETHINLKVSDGSSEIFFKIKKTTPLRRL      60
Recombinant_NSEγ-After-Purification-Tag-Cleavage            ------------------------------------------------------------      0


NSEγ-Internal-Standard_After-Purification-Tag-Cleavage      --------------------------------------------------SIEKIWAREI      10
NSEγ-Internal-Standard_Before-Purification-Tag-Cleavage     --------------------------------------------------SIEKIWAREI      10
Recombinant_NSEγ-Before-Purification-Tag-Cleavage           MEAFAKRQGKEMDSLRFLYDGIRIQADQTPEDLDMEDNDIIEAHREQIGGSIEKIWAREI      120
Recombinant_NSEγ-After-Purification-Tag-Cleavage            --------------------------------------------------SIEKIWAREI      10
                                                                                                              **********

NSEγ-Internal-Standard_After-Purification-Tag-Cleavage      LDSRGNPTVEVDLYTAKGLFRAAVPSGASTGIYEALELRDGDKQRYLGKGVLKAVDHINS      70
NSEγ-Internal-Standard_Before-Purification-Tag-Cleavage     LDSRGNPTVEVDLYTAKGLFRAAVPSGASTGIYEALELRDGDKQRYLGKGVLKAVDHINS      70
Recombinant_NSEγ-Before-Purification-Tag-Cleavage           LDSRGNPTVEVDLYTAKGLFRAAVPSGASTGIYEALELRDGDKQRYLGKGVLKAVDHINS      180
Recombinant_NSEγ-After-Purification-Tag-Cleavage            LDSRGNPTVEVDLYTAKGLFRAAVPSGASTGIYEALELRDGDKQRYLGKGVLKAVDHINS      70
                                                            ************************************************************

NSEγ-Internal-Standard_After-Purification-Tag-Cleavage      TIAPALISSGLSVVEQEKLDNLMLELDGTENKSKFGANAILGVSLAVCKAGAAERELPLY      130
NSEγ-Internal-Standard_Before-Purification-Tag-Cleavage     TIAPALISSGLSVVEQEKLDNLMLELDGTENKSKFGANAILGVSLAVCKAGAAERELPLY      130
Recombinant_NSEγ-Before-Purification-Tag-Cleavage           TIAPALISSGLSVVEQEKLDNLMLELDGTENKSKFGANAILGVSLAVCKAGAAERELPLY      240
Recombinant_NSEγ-After-Purification-Tag-Cleavage            TIAPALISSGLSVVEQEKLDNLMLELDGTENKSKFGANAILGVSLAVCKAGAAERELPLY      130
                                                            ************************************************************

NSEγ-Internal-Standard_After-Purification-Tag-Cleavage      RHIAQLAGNSDLILPVPAFNVINGGSHAGNKLAMQEFMILPVGAESFRDAMRLGAEVYHT      190
NSEγ-Internal-Standard_Before-Purification-Tag-Cleavage     RHIAQLAGNSDLILPVPAFNVINGGSHAGNKLAMQEFMILPVGAESFRDAMRLGAEVYHT      190
Recombinant_NSEγ-Before-Purification-Tag-Cleavage           RHIAQLAGNSDLILPVPAFNVINGGSHAGNKLAMQEFMILPVGAESFRDAMRLGAEVYHT      300
Recombinant_NSEγ-After-Purification-Tag-Cleavage            RHIAQLAGNSDLILPVPAFNVINGGSHAGNKLAMQEFMILPVGAESFRDAMRLGAEVYHT      190
                                                            ************************************************************

NSEγ-Internal-Standard_After-Purification-Tag-Cleavage      LKGVIKDKYGKDATNVGDEGGFAPNILENSEALELVKEAIDKAGYTEKIVIGMDVAASEF      250
NSEγ-Internal-Standard_Before-Purification-Tag-Cleavage     LKGVIKDKYGKDATNVGDEGGFAPNILENSEALELVKEAIDKAGYTEKIVIGMDVAASEF      250
Recombinant_NSEγ-Before-Purification-Tag-Cleavage           LKGVIKDKYGKDATNVGDEGGFAPNILENSEALELVKEAIDKAGYTEKIVIGMDVAASEF      360
Recombinant_NSEγ-After-Purification-Tag-Cleavage            LKGVIKDKYGKDATNVGDEGGFAPNILENSEALELVKEAIDKAGYTEKIVIGMDVAASEF      250
                                                            ************************************************************

NSEγ-Internal-Standard_After-Purification-Tag-Cleavage      YRDGKYDLDFKSPTDPSRYITGDQLGALYQDFVRDYPVVSIEDPFDQDDWAAWSKFTANV      310
NSEγ-Internal-Standard_Before-Purification-Tag-Cleavage     YRDGKYDLDFKSPTDPSRYITGDQLGALYQDFVRDYPVVSIEDPFDQDDWAAWSKFTANV      310
Recombinant_NSEγ-Before-Purification-Tag-Cleavage           YRDGKYDLDFKSPTDPSRYITGDQLGALYQDFVRDYPVVSIEDPFDQDDWAAWSKFTANV      420
Recombinant_NSEγ-After-Purification-Tag-Cleavage            YRDGKYDLDFKSPTDPSRYITGDQLGALYQDFVRDYPVVSIEDPFDQDDWAAWSKFTANV      310
                                                            ************************************************************

NSEγ-Internal-Standard_After-Purification-Tag-Cleavage      GIQIVGDDLTVTNPKRIERAVEEKACNCLLLKVNQIGSVTEAIQACKLAQENGWGVMVSH      370
NSEγ-Internal-Standard_Before-Purification-Tag-Cleavage     GIQIVGDDLTVTNPKRIERAVEEKACNCLLLKVNQIGSVTEAIQACKLAQENGWGVMVSH      370
Recombinant_NSEγ-Before-Purification-Tag-Cleavage           GIQIVGDDLTVTNPKRIERAVEEKACNCLLLKVNQIGSVTEAIQACKLAQENGWGVMVSH      480
Recombinant_NSEγ-After-Purification-Tag-Cleavage            GIQIVGDDLTVTNPKRIERAVEEKACNCLLLKVNQIGSVTEAIQACKLAQENGWGVMVSH      370
                                                            ************************************************************

NSEγ-Internal-Standard_After-Purification-Tag-Cleavage      RSGETEDTFIADLVVGLCTGQIKTGAPCRSERLAKYNQLMRIEEELGDEARFAGHNFRNP      430
NSEγ-Internal-Standard_Before-Purification-Tag-Cleavage     RSGETEDTFIADLVVGLCTGQIKTGAPCRSERLAKYNQLMRIEEELGDEARFAGHNFRNP      430
Recombinant_NSEγ-Before-Purification-Tag-Cleavage           RSGETEDTFIADLVVGLCTGQIKTGAPCRSERLAKYNQLMRIEEELGDEARFAGHNFRNP      540
Recombinant_NSEγ-After-Purification-Tag-Cleavage            RSGETEDTFIADLVVGLCTGQIKTGAPCRSERLAKYNQLMRIEEELGDEARFAGHNFRNP      430
                                                            ************************************************************

NSEγ-Internal-Standard_After-Purification-Tag-Cleavage      SVLLVPR------------      437
NSEγ-Internal-Standard_Before-Purification-Tag-Cleavage     SVLLVPRGSHHHHHHHHHH      449
Recombinant_NSEγ-Before-Purification-Tag-Cleavage           SVL----------------      543
Recombinant_NSEγ-After-Purification-Tag-Cleavage            SVL----------------      433
                                                            ***
```

Figure 1 Sequence alignment of recombinant NSEγ and NSEγ internal standard before and after purification tag cleavage.

Page S3. Supporting Information 3 Expressed Protein Characterization LC-UV system

A. Recombinant NSEγ B. NSEγ - Internal Standard

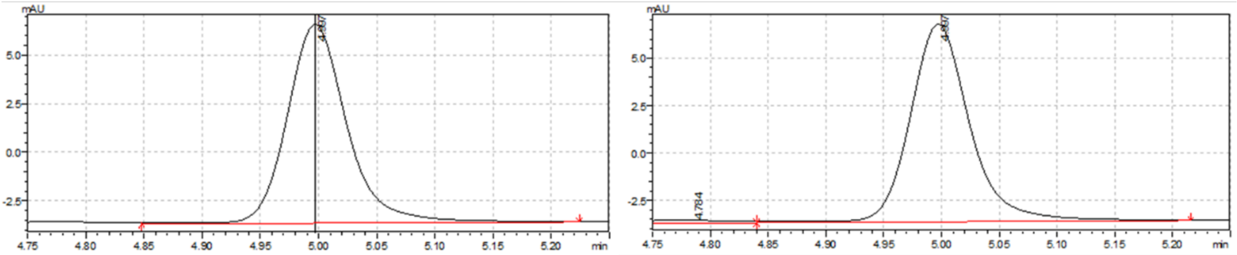

Figure 2 LC-UV Chromatograms of Recombinant NSEγ (A) and NSEγ internal standard (B).

Table 1 Retention time, peak area, and peak height of recombinant NSEγ and NSEγ Internal standard at 100 ng on column using LC-UV.

**Protein**	**Compound on column**	**Retention time (min)**	**Peak Area**	**Peak Height**
A. NSEγ	100 ng	5.0	36789	10210
B. NSEγ-IS	100 ng	5.0	37616	10393

Page S4. Supporting Information 4 Expressed Protein Characterization LC-MS system

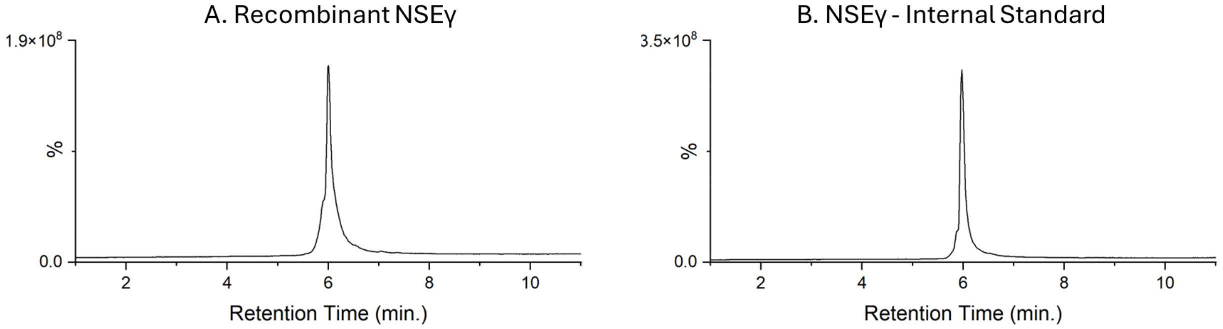

Figure 3 LC-MS Chromatograms of Recombinant NSEγ (A) and NSEγ internal standard (B).

Table 2 Retention time, peak area, and peak height of recombinant NSEγ and NSEγ Internal standard at 100 ng on column using LC-MS.

Protein	Compound on column	Retention time (min)	Peak Area	Peak Height
A. NSEγ	10 ng	6.0	3.5e7	1.5e8
B. NSEγ-IS	10 ng	6.0	3.9e7	2.7e8

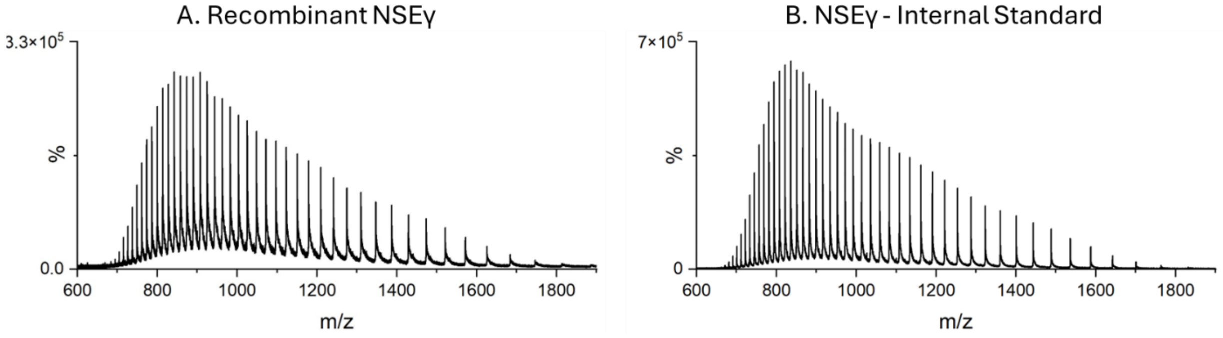

Figure 4 Mass Spectra of Recombinant NSEγ (A) and NSEγ internal standard (B).

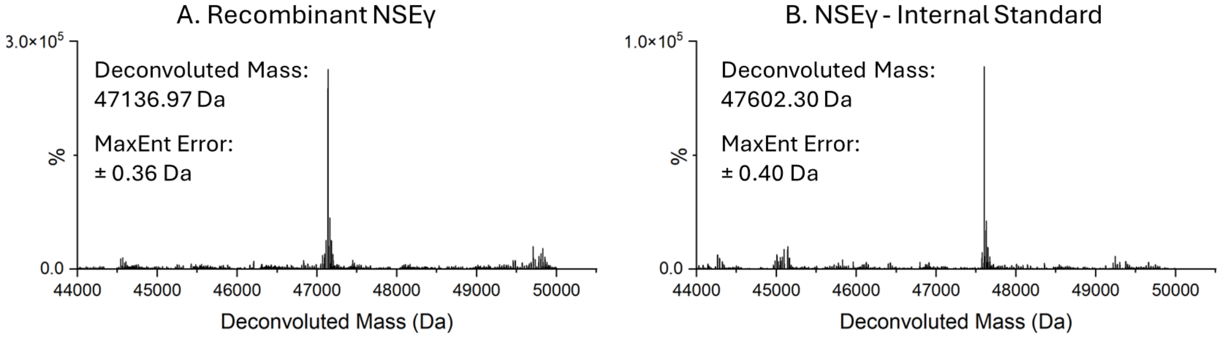

Figure 5 MaxEnt1 deconvoluted mass and MaxEnt Mass errors of Recombinant NSEγ (A) and NSEγ internal standard (B).

Page S5. Supporting Information 5 Matrix Effect

Table 3 Matrix effect tested by spiking six serum samples with 50 ng/mL recombinant NSEγ, followed by subtracting the endogenous NSEγ concentration from the measured un-spiked samples. Displayed are the measured concentrations and bias (%) from 50 ng/mL.

Matrix Sample	Measured (ng/mL)	Bias (%)
1	42.5	-15.0
2	57.9	+9.9
3	44.5	-10.9
4	50.0	0.0
5	42.9	-14.1
6	55.8	+11.7

References

(1) Madeira, F.; Pearce, M.; Tivey, A. R. N.; Basutkar, P.; Lee, J.; Edbali, O.; Madhusoodanan, N.; Kolesnikov, A.; Lopez, R. Search and Sequence Analysis Tools Services from EMBL-EBI in 2022. *Nucleic Acids Res.* **2022**, *50* (W1), W276–W279. https://doi.org/10.1093/nar/gkac240.
